# Sport Commitment among Malaysian Racquet Sports Athletes

**DOI:** 10.21315/mjms2019.26.4.10

**Published:** 2019-08-29

**Authors:** Arthur Ling, Eng Wah Teo, Ngien Siong Chin

**Affiliations:** 1 Sport Centre, University Malaya, Kuala Lumpur, Malaysia; 2 Institute of Teacher Education Batu Lintang, Kuching Sarawak, Malaysia

**Keywords:** constrained commitment, enthusiastic commitment, racquet sports, sport commitment, Sport Commitment Model, sport psychology

## Abstract

**Background:**

The Sport Commitment Model is widely used to understand the motivation and commitment of athletes to continue playing sports. However, the factors influencing athletes’ commitment to racquet sports have not received much research attention, especially in Malaysia.

**Purpose:**

This study aims to use the Sport Commitment Questionnaire-2 (SCQ-2) to examine Malaysian athletes’ commitment to racquet sports.

**Methods:**

A total of 612 athletes (367 males/245 females, *μ* age= 30.32 ± 11.56) completed the SCQ-2, which measures seven factors and two dimensions of sport commitment.

**Results:**

The results revealed that sport enjoyment was the main factor contributing to the athletes’ commitment in all sports. Two-way ANOVA analyses showed significant differences in athletes’ enthusiastic commitment [*F*(_3,604_) = 44.92, *P* = 0.00] and constrained commitment [*F*_(3,604)_ = 15.32, *P* = 0.00] across four sports. There were also significant differences in both enthusiastic commitment [*F*(_3,604_) = 7.53, *P* = 0.00] and constraint commitment [*F*(_3,604_) = 18.82, *P* = 0.00] across age groups.

**Conclusion:**

Enjoyment is the main factor in sport commitment. Tennis athletes possess the highest level of enthusiastic commitment across all the racquet sports. Moreover, male athletes showed higher levels of enthusiastic commitment than female athletes.

## Introduction

Sports commitment is defined as the willingness to remain active in sports activities (1). Currently, one of the most popular models in understanding athletes’ desire to continue participating in sports is the Sport Commitment Model (SCM) developed by Scanlan et al. (1).

Over the past 20 years, SCM has been used to examine the factors predicting sport commitment in various sports across countries. Carpenter and Scanlan (2) reported that enjoyment was the main predictor of sport commitment among 103 US soccer players. Weiss et al. (3) also reported a similar result in that enjoyment was the primary predictor of sport commitment among 198 US junior tennis athletes. Subsequently, Weiss and Weiss (4) found that the commitment of 124 US female gymnasts was governed by the level of enjoyment in training and competition. In another study on adult US tennis players, Casper et al. (5) revealed that enjoyment and personal investment were the most influential predictors of sport commitment. In 2008, Casper and Stellino (6) evaluated 537 US recreational tennis athletes and found that enjoyment was the strongest predictor of sport commitment. Chairat et al. (7) also found that the Thai version of the Athlete Opinion Survey, administered to 460 Thai athletes, showed SCM to be valid and reliable. In addition, Wigglesworth et al. (8) found that enjoyment was the main predictor of sport commitment for 507 masters/senior level swimmers from 37 countries. Similar findings have been reported in a study on 352 US soccer players conducted by Frayeh and Lewis (9). Therefore, enjoyment has consistently emerged as the main predictor of sport commitment regardless of performance or recreational level. However, Alexandris et al. (10) conducted a study of 210 health club members in Greece and reported that opportunities for involvement were the main predictor of sport commitment.

From a different commitment perspective, i.e., sport commitment and gender, Casper and Stellino (6) evaluated 539 US recreational adult tennis players and reported that female tennis athletes perceived slightly higher enjoyment and personal investment compared to their male counterparts, but there was no significant difference across gender. They also revealed that male tennis players received higher social support as compared to female players (6). Similar to Casper and Stellino’s (6) finding, Jess’s (11) study on 302 Canadian university students also found there was no significant difference in sport commitment across gender. In addition, Boyst (12) also reported that there was no gender difference in sport commitment among 101 US collegiate soccer athletes. However, Wigglesworth et al. (8) revealed that senior male swimmers showed higher enthusiastic commitment compared to senior female swimmers, which indicated that male swimmers were more willing to continue their participation in swimming. Wigglesworth et al. (8) also revealed that senior male swimmers made a greater personal investment than senior female swimmers, i.e., male swimmers were spending more money to take part in competitions. Thus, it can be said that male and female athletes perceived sport enjoyment as the most important predictor to sport commitment. However, the results of previous studies regarding sports commitment and gender are inconclusive.

In addition to gender differences, some studies (5, 13, 14) also looked into age factors on sports commitment. Casper et al. (5) revealed that older tennis athletes (above 45 years old) were more committed than younger tennis athletes (19–44 years old) in the US. In another study, Weiss and Neibert (13) reported that the level of athletes’ commitment may change as athletes grow older. For example, US athletes at the graduate level showed a lower level of commitment toward sports as compared to those at the undergraduate level because they no longer experienced the same sense of enjoyment. In a comparison of sport commitment between US collegiate athletes versus high school athletes, Weiss (14) revealed that these two age groups differed in that collegiate athletes had a higher perception of investments, costs, opportunities, perceived competence, social support and performance-motivational climate than high school athletes. Meanwhile, for high school athletes, perceived social constraints and mastery-motivational climate were the important predictors of sport commitment.

Drawing on the line of research noted above, this study aims to use Sport Commitment Questionnaire-2 (SCQ-2) to examine the factors that influence the sport commitment of racquet sport athletes (i.e., badminton, table tennis, tennis and squash). Secondly, we aim to evaluate the effect of types of sports and gender on athletes’ commitment and the relationship between age groups and gender of racquet sports athletes’ commitment.

## Methods

### Research Design

This study is a cross-sectional study design whereby athletes from four racket sports were recruited from across Malaysia.

### Participants and Sampling

A total of 612 Malaysian athletes (367 males, 245 females) comprising 155 badminton athletes, 152 table tennis athletes, 153 tennis athletes and 152 squash athletes and ranging in age from 18–65 years (*μ* = 30.32 ± 11.56 years) participated in the study. The sample size was determined using G-power 3.0.10 based on multiple regression. The estimated sample size for each sport was 151, given the effect size of 0.10, alpha of 0.05, and power of 0.80. Stratified sampling was used to identify the proportions needed for the study.

### Instrument

The SCQ-2 was administered to the racquet sport athletes. The questionnaire consists of two parts: demographic questions and SCQ-2. Demographic information such as gender, age and type of sport played were collected.

The SCQ-2 consists of 58 items based on a five-point Likert scale ranging from 1 = strongly disagree to 5 = strongly agree. Its overall reliability with seven subscales was above 0.80 and composite reliabilities ranged from 0.71 to 0.92. Furthermore, the SCQ-2 showed good validity and reliability in the sports setting [χ^2^(1530) = 3327.33, *P* < 0.001, NNFI = 0.89, CFI = 0.90, SRMR = 0.04, RMSEA = 0.04] (15).

In addition, a pilot study was administered in Malaysia to athletes playing racquet sports to ensure the suitability of the instrument and identify issues that might arise during the data collection process. During that process, the researcher had observed the ways in which the participants answered the questions and clarified items as necessary. A total of 50 questionnaires were collected during the pilot test. The Cronbach’s alphas for the seven factors were sport enjoyment, α = 0.85; other priorities, α = 0.88; personal investments, α = 0.92; social constraints, α = 0.90; valuable opportunities, α = 0.87; social support, α = 0.94; and desire to excel; α = 0.93. Furthermore, the two dimensions of commitment comprised of enthusiastic commitment (α = 0.89) and constrained commitment (α = 0.86). The overall Cronbach’s alpha score was 0.85 when tested with Malaysian players of racquet sports.

### Procedures

Prior to data collection, ethics approval was obtained from University Malaya Ethics Board (UM. TCN2/RCH&E/UMREC-14). The details regarding the research, such as the objective and nature of the questionnaire, were clearly explained to the athletes prior to their signing the consent form, which was an indication of their voluntary participation. The participants were informed that they had the right to withdraw from the study at any time and without any consequences. There were 900 questionnaires distributed throughout the country, and 654 (72.6%) of those questionnaires were returned. However, during data cleaning, 42 questionnaires were removed from the data set because of missing data.

### Data Analysis

SPSS Statistics version 23.0 was used to perform all statistical analyses in this study. Descriptive statistics, two-way ANOVA and post-hoc Bonferroni were computed to address all the research questions of this study.

## Results

Table 1 shows the demographic information of the players involved in this study. A total of 612 athletes took part in this study (367 males/245 females, *μ* age= 30.32 ± 11.56), and they represented 155 badminton athletes, 152 table tennis athletes, 153 tennis athletes and 152 squash athletes.

Table 2 revealed that enjoyment was the main predictor of sport commitment for all racquet sports in Malaysia, and the mean score of sport enjoyment for badminton *μ* = 4.38 ± 0.51, table tennis *μ* = 4.37 ± 0.54, tennis *μ* = 4.79 ± 0.50 and squash *μ* = 4.62 ± 0.47.

Enthusiastic commitment and constraint commitment were analysed for all four (sports: badminton, table tennis, tennis and squash) × two (gender: male versus female) between-subjects ANOVA. The main effect of sports on enthusiastic commitment was significant [*F*(3,604) = 44.92, *P* = 0.00], and the main effect of sports on constraint commitment was also significant [*F*(3,604) = 15.32, *P* = 0.00]. The additional post-hoc Bonferroni tests showed that enthusiastic commitment differed significantly between badminton versus tennis (*P* = 0.00, *d* = −0.13, effect size is small); badminton versus squash (*P* = 0.00, *d* = −1.00, effect size is large); table tennis versus tennis (*P* = 0.00, *d* = 0.80, effect size is large); table tennis versus squash (*P* = 0.00, *d* = 0.69, effect size is medium). Badminton rated 0.66 points lower in enthusiastic commitment than tennis (*P* = 0.00, 95% confidence interval (CI) of the difference = −0.84 to −0.49) and 0.57 points lower than squash (*P* = 0.00, 95% CI of the difference = −0.75 to −0.39). In contrast, table tennis rated 0.53 points lower in enthusiastic commitment than tennis (*P* = 0.00, 95% CI of the difference = −0.84 to −0.49) and 0.44 points lower than squash (*P* = 0.00, 95% CI of the difference = −0.62 to −0.26). However, Table 3 also revealed significant differences for constrained commitment between badminton and tennis (*P* = 0.00, *d* = 0.59, effect size is medium], table tennis versus tennis (*P* = 0.00, *d* = 0.80, effect size is large], and squash versus tennis (*P* = 0.00, *d* = 0.53, effect size is medium]. Post-hoc Bonferroni tests showed that badminton rated 0.45 points higher in constrained commitment than tennis (*P* = 0.00, 95% CI of the difference = −0.19 to 0.71). In comparison, tennis rated 0.69 points lower in constrained commitment than table tennis (*P* = 0.00, 95% CI of the difference = 0.43 to 0.95) and 0.49 points lower than squash (*P* = 0.00, 95% CI of the difference = −0.763 to −0.23).

The main effect of gender was only significant for enthusiastic commitment [*F*(1,604) = 20.54, *P* = 0.00] but not for constraint commitment [*F*(1,604) = 1.25, *P* = 0.19]. Table 4 revealed that Bonferroni-adjusted comparisons for enthusiastic commitment indicated that male badminton athletes rated 0.22 points higher than female badminton athletes (*P* = 0.02, 95% CI of the difference = 0.03 to 0.40), male table tennis athletes rated 0.36 points higher than their female counterparts (*P* = 0.00, 95% CI of the difference = 0.16 to 0.55) and male tennis athletes also rated 0.21 points higher than female tennis athletes (*P* = 0.04, 95% CI of the difference = 0.01 to 0.41). In contrast, the ratings of male and female did not differ significantly for squash (*P* = 0.30). There was no significant interaction between sports and gender for both enthusiastic commitment [*F*(3,604) = 1.18, *P* = 0.32] and constraint commitment [*F*(3,604) = 0.26, *P* = 0.85].

Enthusiastic commitment and constraint commitment were analysed with four (age groups: 11–20, 21–30, 31–40 and > 40) × two (gender: male versus female) between-subjects ANOVA. The main effect of age group was both significant on enthusiastic commitment [*F*(3,604) = 7.53, *P* = 0.00] and constraint commitment [*F*(3,604) = 18.82, *P* = 0.00]. A post-hoc test for enthusiastic commitment shown in Table 5 revealed that there were significant differences between age groups 11–20 years versus 31–40 years (*P* = 0.01, *d* = 0.44, effect size is small), 11–20 years versus > 40 years (*P* = 0.00, *d* = 0.47, effect size is small), 21–30 years versus 31–40 years (*P* = 0.01, *d* = 0.41, effect size is small) and 21–30 years versus > 40 years (*P* = 0.00, *d* = 0.37, effect size is small). For constrained commitment, the results showed that there was a significant difference between age groups 11–20 years versus 31–40 years (*P* = 0.00, *d* = 0.81, effect size is large), 11–20 years versus > 40 years (*P* = 0.00, *d* = 0.69, effect size is medium), 21–30 years versus 31–40 years (*P* = 0.00, *d* = 0.56, effect size is medium), and 21–30 years versus > 40 years (*P* = 0.00, *d* = 0.44, effect size is small). The only significant difference was found in the main effect of gender on enthusiastic commitment [*F*(1,604) = 11.20, *P* = 0.00]. Table 6 revealed Bonferroni-adjusted comparisons indicated that age group 11–20 years rated the male athletes 0.28 points higher in enthusiastic commitment than the female athletes (*P* = 0.01, 95% CI of the difference = 0.08 to 0.48), while age group 21–30 years rated the male athletes 0.24 points higher in enthusiastic commitment than the female athletes (*P* = 0.01, 95% CI of the difference = 0.06 to 0.41). There was no significant interaction between age group and gender for both enthusiastic commitment [*F*(3,604) = 0.80, *P* = 0.49] and constraint commitment [*F*(3,604) = 1.10, *P* = 0.35].

## Discussion

Our results revealed that the factor of enjoyment was the main influence on racquet athletes’ commitment among Malaysians. Therefore, this result is in line with previous studies by Baghurst et al. (16); Carpenter et al. (17); Chairat et al. (7); Weiss and Weiss, (4); Weiss and Neibert (13); Wigglesworth et al. (8); and Iñigo et al. (18). In general, Malaysian racquet sport athletes were passionate and have positive feelings towards their chosen sports (19).

Among the athletes engaged in the four racquet sports, tennis athletes showed the highest level of enthusiastic commitment (*μ* = 4.41 ± 0.48) and the lowest level of constrained commitment (*μ* = 1.79 ± 0.80). Our results are similar to Casper and Stellino’s (6) study that reported sport enjoyment to be the most influential predictor of tennis athletes’ commitment as they did not feel the pressure that indirectly lowered their constrained commitment (6). Badminton players showed the lowest enthusiastic commitment (*μ* = 3.74 ± 0.62) relative to other racquet sports. The result was unexpected because in Malaysia, after football, badminton is the most popular sport in Malaysia.

According to Scanlan et al. (20), one of the factors that contribute to enthusiastic commitment is the ‘desire to excel’. In our study, badminton athletes scored the lowest in terms of ‘desire to excel’ (*μ* = 3.74 ± 0.67) when compared to athletes of other racquet sports (Table 3). One reason may be because the majority of the badminton players in our study were pursuing the sport at the recreational level and as a social activity without the expressed goal of excelling at this sport. Thus, this could explain why badminton showed low levels of enthusiastic commitment.

The results from two-way ANOVA analyses confirmed a significant positive relationship between enthusiastic commitment in sports and gender. This suggests that male athletes, when compared to female ones, normally have higher levels of enthusiastic commitment. The reason why female athletes have lower levels of sport commitment could be due to the lack of social support from friends, family or spouse (21, 22). Our findings also revealed that female athletes showed higher constrained commitment than male athletes. One reason might be that females prioritise things other than sports such as families and careers, leading to their tendency to abandon sports.

From the perspective of age group, our result also showed that athletes from younger age groups (11–20 years and 21–30 years), which include university students and young adults, perceived higher constrained commitment than other age groups. This could be due to external pressure on them to secure scholarships and a future athletic career (12). The results generally support Casper and Stellino’s (6) study stating that younger tennis players experience greater social constraints than older tennis athletes. Here, the impact of greater social constraints might lead to greater constraints commitment. Interestingly, the older age groups (31–40 years and above 40 years) have the highest levels of enthusiastic commitment, higher than those of the younger age groups. The reason could be that these older athlete groups, who may hold senior positions in their respective companies, might be experiencing a stressful time of their career.

This study has several limitations. First, in Malaysia, the proportion of elite and non-elite players remains unknown because no data has been collected for players of racquet sports in Malaysia. To overcome this problem, G-power was used to calculate the sample size needed for this study (refer ‘participants’). Second, some Malaysians, especially those from rural areas who answered this questionnaire, are more fluent in Bahasa Malaysia or other native Malaysian languages while the SCQ-2 is in English. Hence, some of their feedback might not fully reflect what they feel. To overcome this limitation, the players were assisted by research assistants when they need to clarify items in the questionnaire.

## Conclusion

In conclusion, enjoyment was the main factor contributing to the athletes’ commitment to all racquet sports. The results also showed that tennis players had higher levels of enthusiastic commitment across all racquet sports. In addition, male athletes showed higher levels of enthusiastic commitment compared to females in all racquet sports and across age groups. Therefore, it is important for coaches and sports administrators in any country to understand these differences in commitment. In addition to lowering the burn-out or drop-out rate, valuing effort, training and success can lead to athletes showing a stronger level of commitment and encourage their positive growth

## Figures and Tables

**Table 1:** Socio-demographic characteristic of athletes

Variables	*N*	Percentage (%)
Gender	612	100
Male	367	60
Female	245	40
Age of group (years)		
11–20	157	25.7
21–30	212	34.6
31–40	106	17.3
> 40	137	22.4
Location		
Peninsular Malaysia	330	53.9
Sabah & Sarawak	242	39.5
Wilayah Persekutuan	40	6.5
Sports		
Badminton	155	25.3
Table Tennis	152	24.8
Tennis	153	25.0
Squash	152	24.8

**Table 2:** Descriptive analysis of subscales of racquet sports

Subscales	Badminton (*n* = 155)		Table Tennis (*n* = 152)		Tennis (*n* = 153)		Squash (*n* = 152)	
			
	Mean	SD	Mean	SD	Mean	SD	Mean	SD
Sources								
Sport enjoyment	4.38	0.51	4.37	0.54	4.79	0.34	4.62	0.47
Valuable opportunities	3.24	0.74	3.48	0.82	3.17	0.68	3.63	0.82
Other priorities	2.62	0.87	2.81	0.88	2.06	0.83	2.80	1.03
Personal investment	3.17	0.61	3.38	0.69	3.23	0.55	3.63	0.68
Social constraints	2.75	0.72	2.97	0.88	2.64	0.76	3.31	0.79
Social support	3.07	0.70	3.23	0.81	2.82	0.74	3.47	0.76
Desire to excel	3.48	0.67	3.70	0.64	3.62	0.73	3.97	0.68
Dimension								
Enthusiastic commitment	3.74	0.62	3.87	0.70	4.41	0.48	4.31	0.56
Constrained commitment	2.24	0.71	2.48	0.92	1.79	0.80	2.28	0.96

Scale: 1- strongly disagree, 3- neither agree nor disagree, 5- strongly agree

**Table 3:** Post-hoc Bonferroni test of enthusiastic commitment and constrained commitment between sports

Commitment	*μ* Difference	*P*	95% CI of the difference	

			Lower bound	Upper bound
Enthusiastic commitment				
B versus TT	−0.13	0.31	−0.31	0.05
B versus T	−0.66	0.00	−0.84	−0.49
B versus S	−0.57	0.00	−0.75	−0.39
TT versus T	−0.53	0.00	−0.71	−0.35
TT versus S	−0.44	0.00	−0.62	−0.26
T versus S	0.09	1.00	−0.09	0.27
Constrained commitment				
B versus TT	−0.24	0.08	−0.50	0.02
B versus T	0.45	0.00	0.19	0.71
B versus S	−0.04	1.00	−0.30	0.22
TT versus T	0.69	0.00	0.43	0.95
TT versus S	0.20	0.22	−0.05	0.46
T versus S	−0.49	0.00	−0.76	−0.23

Note: B = badminton, TT = table tennis, T = tennis, S = squash

**Table 4:** Pairwise comparison of male and female enthusiastic commitment in each sport

Enthusiastic Commitment		*μ* Difference	*P*	95% CI of the difference	
	
Sport	Gender			Lower bound	Upper bound
Badminton	Male versus Female	0.22	0.02	0.03	0.40
Table tennis	Male versus Female	0.36	0.00	0.16	0.55
Tennis	Male versus Female	0.21	0.04	0.01	0.41
Squash	Male versus Female	0.10	0.30	−0.09	0.29

**Table 5:** Post-hoc Bonferroni test of enthusiastic commitment and constrained commitment between age groups

Commitment	*μ* Difference	*P*	95% CI of the difference	

			Lower bound	Upper bound
Enthusiastic commitment				
1 versus 2	−0.06	1.00	−0.24	0.12
1 versus 3	−0.31	0.01	−0.53	−0.10
1 versus 4	−0.30	0.00	−0.50	−0.10
2 versus 3	−0.26	0.01	−0.46	−0.05
2 versus 4	−0.24	0.00	−0.43	−0.06
3 versus 4	0.01	1.00	−0.21	0.23
Constrained commitment				
1 versus 2	0.21	0.12	−0.03	0.45
1 versus 3	0.67	0.00	0.38	0.95
1 versus 4	0.58	0.00	0.32	0.84
2 versus 3	0.46	0.00	0.19	0.73
2 versus 4	0.37	0.00	0.13	0.62
3 versus 4	−0.09	1.00	0.38	0.20

Note: 1 = 11–20 years old; 2 = 21–30 years old; 3 = 31–40 years old; 4 = > 40 years old

**Table 6:** Pairwise comparison of male and female enthusiastic commitment in each age group

Enthusiastic commitment		*μ* Difference	P	95% CI of the difference	
	
Age group	Gender			Lower bound	Upper bound
11–20	Male versus Female	0.28	0.01	0.08	0.48
21–30	Male versus Female	0.24	0.01	0.06	0.41
31–40	Male versus Female	0.20	0.13	−0.06	0.45
> 40	Male versus Female	0.04	0.75	−0.20	0.28
